# Automation of a Nile red staining assay enables high throughput quantification of microalgal lipid production

**DOI:** 10.1186/s12934-016-0433-7

**Published:** 2016-02-09

**Authors:** Holger Morschett, Wolfgang Wiechert, Marco Oldiges

**Affiliations:** Forschungszentrum Jülich GmbH, Institute of Bio- and Geosciences, IBG-1: Biotechnology, Wilhelm-Johnen-Straße, 52428 Jülich, Germany

**Keywords:** *Chlorella vulgaris*, High throughput, Laboratory automation, Lipid quantification, Liquid handling robot, Nile red

## Abstract

**Background:**

Within the context of microalgal lipid production for biofuels and bulk chemical applications, specialized higher throughput devices for small scale parallelized cultivation are expected to boost the time efficiency of phototrophic bioprocess development. However, the increasing number of possible experiments is directly coupled to the demand for lipid quantification protocols that enable reliably measuring large sets of samples within short time and that can deal with the reduced sample volume typically generated at screening scale. To meet these demands, a dye based assay was established using a liquid handling robot to provide reproducible high throughput quantification of lipids with minimized hands-on-time.

**Results:**

Lipid production was monitored using the fluorescent dye Nile red with dimethyl sulfoxide as solvent facilitating dye permeation. The staining kinetics of cells at different concentrations and physiological states were investigated to successfully down-scale the assay to 96 well microtiter plates. Gravimetric calibration against a well-established extractive protocol enabled absolute quantification of intracellular lipids improving precision from ±8 to ±2 % on average. Implementation into an automated liquid handling platform allows for measuring up to 48 samples within 6.5 h, reducing hands-on-time to a third compared to manual operation. Moreover, it was shown that automation enhances accuracy and precision compared to manual preparation. It was revealed that established protocols relying on optical density or cell number for biomass adjustion prior to staining may suffer from errors due to significant changes of the cells’ optical and physiological properties during cultivation. Alternatively, the biovolume was used as a measure for biomass concentration so that errors from morphological changes can be excluded.

**Conclusions:**

The newly established assay proved to be applicable for absolute quantification of algal lipids avoiding limitations of currently established protocols, namely biomass adjustment and limited throughput. Automation was shown to improve data reliability, as well as experimental throughput simultaneously minimizing the needed hands-on-time to a third. Thereby, the presented protocol meets the demands for the analysis of samples generated by the upcoming generation of devices for higher throughput phototrophic cultivation and thereby contributes to boosting the time efficiency for setting up algae lipid production processes.

**Electronic supplementary material:**

The online version of this article (doi:10.1186/s12934-016-0433-7) contains supplementary material, which is available to authorized users.

## Background

To promote the shift from fossil resources to a sustainable bio-economy, microalgal production of lipids for biofuels and bulk chemical applications have been intensively investigated for the last two decades [1, 2]. By focusing on the establishment of parallelized microscale cultivation techniques for higher throughput, this research strives towards enabling the screening of algal strain libraries or the investigation of comprehensive sets of process parameters within a reasonable time-scale. Therefore, the use of microtiter plates (MTPs) is one promising approach [3–8]. In this context, appropriate lipid quantification protocols become indispensable that enable measuring large sets of samples in sufficient replicates within short time. Moreover, they must be able to deal with small sample volumes as generated from microscale cultivation level.

Apart from elaborated, but highly expensive methods like time-domain nuclear magnetic resonance and liquid or gas chromatography coupled to mass spectrometry [9, 10], gravimetric methods are the best-established methods for lipid quantification. They mostly rely on two-phase chloroform–methanol/water extraction of biomass and subsequent gravimetric measurement of the relative cellular lipid fraction [11–15]. Despite the significant progress regarding protocol simplification [16], extractive lipid quantification still remains unsuitable for microscale high throughput applications for the following reasons: (1) Solvent extraction and gravimetry are multistep procedures implying significant time effort for the handling of consumables (drying, weighing) and solvent evaporation. Even the fastest protocols need at least 3 days, containing a large proportion of hands-on-time and thereby significantly limiting experimental throughput. (2) At least 20 mg of biomass are needed for reliable analysis. This amount cannot be generated from MTP cultivations at millilitre-scale. Especially during early growth phase, only very limited amounts of biomass (<0.1 mg) can be harvested from individual wells of an MTP. (3) Quantitative two-phase solvent extraction suffers from high sensitivity regarding fluctuations of process conditions [17]. To conclude, a substantial number of systematic and stochastic errors are expected originating from individual steps of the analytical process of gravimetric lipid quantification. Hence, assessment of relevant errors and error handling is an important issue when down-scaling analytical procedures.

Fluorescent dyes offer an indirect alternative for the detection of intracellular lipids that, on the one hand requires only simple optical measurement and on the other hand enables high throughput applications. Most often, the lipophilic dyes Nile red (9-diethylamino-5-benzo [α] phenoxazinone) and BODIPY^®^ 505/515 (4,4-difluoro-1,3,5,7-tetramethyl-4-bora-3a,4a-diaza-s-indacene) are used [10]. In general, the fluorescence of stained cells is accessible by spectrofluorometry, the use of fluorescence microscopy or flow cytometry providing the possibility to investigate population heterogeneity in lipid content across individual cells from a distinct culture [18]. Spectrofluorometric methods can easily be transferred to MTP scale, thereby enabling automation by using liquid handling robots [10]. BODIPY^®^ staining became increasingly widespread within the last decade due to advantages like superior photostability [19] or fast cell wall permeation and staining kinetics [20]. Nevertheless, Nile red remains the more suitable dye for spectrofluorometric applications in terms of quantitative analysis, since BODIPY^®^ staining suffers from strong background fluorescence of the dye, distorting absolute lipid quantification [19].

Nile red is used as a probe for intracellular lipids since the 1980s [21, 22]. Despite showing poor fluorescence in water, it shows strong fluorescence in hydrophobic environments. However, the fluorescence properties with respect to excitation and emission characteristics, as well as quantum yields are highly dependent on the polarity of the dye’s microenvironment [23]. Thereby, variation of the excitation and emission wavelengths was used to stain different classes of hydrophobic molecules [24–29]. By now, a large number of protocols using Nile red for the quantification of intracellular lipids have been developed, while especially dye permeation was identified as a major bottleneck. Although appropriate staining conditions (cell and dye concentration, temperature, improved permeation via organic solvents or physical pretreatment) have been established for a variety of different microalgae, their applicability remains strain specific so that individual gravimetric calibration is needed to allow for cross-strain comparison [10].

Within this context, this study aims at revealing the potential of Nile red staining for high throughput analysis, introducing a protocol that uses laboratory automation via a liquid handling robot to enable reproducibly measuring large sets of samples with small volume available and minimized hands-on-time. The model organism *Chlorella vulgaris* was used as a biological reference system. It is one of the most representative strains of microalgae and a well-established organism for phototrophic lipid production studies [1, 30–32].

## Results and discussion

### Standardization of biomass concentration

Practically all published methods using spectrofluorometry take place at a constant biomass concentration as the amount of biomass to be stained per assay is directly correlated to the corresponding fluorescence signal. Biomass determination and adjustment are typically done by means of either optical density or cell number for reasons of simplicity [19, 27, 33–36].

**Fig. 1:**
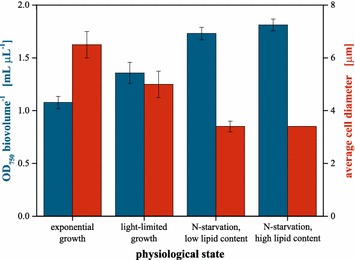
Biovolume-specific optical density and average cell size of *C. vulgaris* at different physiological states. Depending on the physiological state observed, the cells significantly differ in their optical properties and cell size. Thereby, biomass determination across different states by either optical signals or cell number is distorted. Cell samples originated from phototrophic shaking flask cultivations. *Error bars* deviated from samples (biological replicates) of the respective fermentation phases (n ≥ 3)

The model organism *C.vulgaris* used throughout this study replicates via vegetative autosporulation [32, 37] and thereby undergoes significant morphological changes during a cultivation process. As the optical properties of suspended particles are highly dependent on their size, shape and refractive indices [38], the impact on optical density assisted biomass quantification needs to be evaluated. For this purpose, samples of *C.vulgaris* at different physiological states typically occurring during phototrophic fermentation were analysed with regard to their optical properties, measured in terms of simple optical density in a spectrophotometer, as well as cell size and biovolume, making use of particle counting technology (Fig. 1). In this context, the biovolume given in µL_cytoplasm_ mL_sample_^−1^ represents the cytoplasmic volume of the cells per volume of sample. Thus, it is a valid equivalent of the intracellular biological reaction space and can be applied as a measure for biomass concentration (see “Biomass detection” Section for a detailed description of the measurement principle).

The ratio of optical density to biomass (by means of biovolume) was used as a measure for the biomass specific change of the cells’ optical properties. For *C.vulgaris* cells at the different physiological states, it increased significantly (p < 0.05) by more than 60 % from 1.08 ± 0.06 for exponentially growing via 1.36 ± 0.1 for light-limited growing cells to 1.73 ± 0.06 and 1.81 ± 0.05 for N-starved cells containing low and high amounts of intracellular lipids, respectively. In parallel, the average cell size shrank from approximately 6.5 µm during exponential growth down to 3.4 µm during N-starvation.

The changes of the biomass specific optical signal were most likely caused due to morphology dependent light scattering characteristics of the cells. Cell sizes largely differed across the physiological states as the relative fractions of small autospores, replicating mother cells and starved cells varies from non-limited growth to light and subsequently nutrient starvation. Additionally, the composition of *C.vulgaris* cell wall is known to undergo major restructuration during cell cycle [39] which may change the optical properties, as well. Probably, even the accumulation of intracellular lipid droplets (liposomes) [19, 40] might significantly influence the optical properties through a shift of the cellular refraction index.

Consequently, neither optical density, nor cell number can be applied as a valid measure for biomass adjustment across different morphological states and lipid contents. Assuming that the optical properties of the cell, determined by optical density, would show no conflict with exact biomass determination, a constant ratio of OD_750_ to biovolume would have been observed in Fig. 1 (blue bars).

Nevertheless, optical density is frequently used for biomass quantification prior to Nile red staining [27, 33–36] which has to be evaluated quite critically. Instead, the biovolume was chosen as a more reliable measure of biomass to be used for assay development. Its quantification via a particle counter is independent from the cells’ optical properties (size, scattering and absorption characteristics) and can be assessed rather quickly. Being equivalent to the cytoplasmic volume per volume of sample, it represents the amount of biological reaction space available to catalyse lipid synthesis and can thereby be seen as an appropriate parameter for biomass standardization.

### Assay development and validation

The general concept of the Nile red assay to be used was adopted from literature. A spectrofluorometric MTP-based protocol with high potential for automation via a liquid handling robot was chosen as a starting point [33]. Following the optimization experiments given, a dye concentration of 1 mg L^−1^ and an incubation temperature of 40 °C were applied. Staining was conducted in 25 % (v v^−1^) dimethyl sulfoxide (DMSO) to facilitate dye penetration as commonly applied for Nile red staining.

**Fig. 2:**
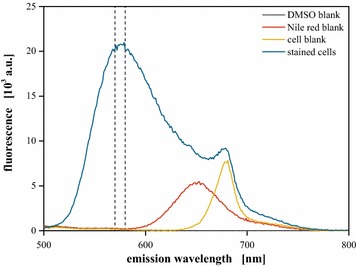
Typical fluorescence spectra of the lipid assay. Staining of cells with the lipid probe Nile red and subsequent excitation at 480 nm results in a specific, lipid correlated fluorescence with a maximum at 570–580 nm. Biomass with a biovolume of 0.15 µL mL^−1^ was stained for 3 h at 40 °C in 25 % (v v^−1^) DMSO with 1 mg L^−1^ Nile red, excitation at 480 nm

Choosing an excitation wavelength of 480 nm for specific detection of neutral lipids, the resulting fluorescence spectrum was evaluated for possible interference by background originating from assay compounds, basal fluorescence of dye and cellular autofluorescence (Fig. 2).

In accordance with literature [26, 33], intracellular neutral lipids could be detected by staining of lipid producing cells via a fluorescence signal with a maximum at 570–580 nm. Neither significant background fluorescence nor Nile red (600–750 nm) or chlorophyll mediated cellular autofluorescence (650–750 nm) were observed to interfere with the lipid signal, so that the average fluorescence signal within the interval of 570–580 nm was chosen as a marker for intracellular lipids.

**Fig. 3:**
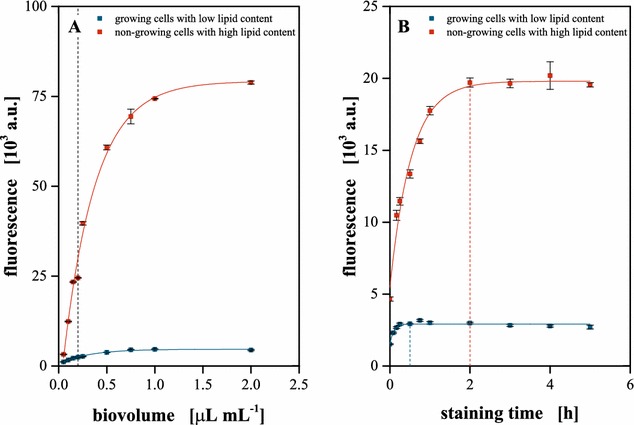
Characterization of the Nile red staining. **a** Biomass specific lipid fluorescence. After an initial linear correlation, the fluorescence signal saturates above a biovolume of 0.2 µL mL^−1^ for both cell types (*dashed line*). **b** Time-dependent staining kinetics of cells with a biovolume of 0.15 µL mL^−1^. In contrast to staining growing cells containing low levels of lipids (0.5 h), non-growing cells with high lipid content need significantly more time (2 h) for quantitative staining. Cells were stained in 25 % (v v^−1^) DMSO containing 1 mg L^−1^ Nile red at 40 °C, excitation at 480 nm. *Error bars* deviated from analytical replicates (n = 5)

As stated previously, conditions for reliable quantitative staining enabling lipid quantification via Nile red fluorescence were observed to be highly strain specific due to varying dye uptake characteristics [10]. Consequently, biomass concentration and staining time were optimized for *C. vulgaris* at the selected dye (1 mg L^−1^) and solvent concentration [25 % (v v^−1^)] and temperature (40 °C), respectively. Non-growing cells with high, as well as growing cells with low lipid content, were used to investigate potentially different staining characteristics while biomass concentration was adjusted by means of biovolume as discussed within the “Standardization of biomass concentration” Section (Fig. 3).

The fluorescence signal deviated from staining was observed to be linearly correlated to the applied cell concentration up to a biovolume of 0.2 µL mL^−1^ for both cell types investigated, while staining of higher biomass concentrations resulted in a signal saturation (Fig. 3a). This could be a consequence of a negative effect in the cell permeation or the staining reaction and thus pointing to a potential limitation of the dye transport or depletion of Nile red at higher biomass concentrations. Especially the latter hypothesis could be explained by adsorption of Nile red to further hydrophobic compartments like membrane phospholipids, thereby reducing the amount of dye available for storage lipid staining.

Regarding the staining kinetics, a diverse pattern was observed. Growing cells, containing only low amounts of neutral lipids were quantitatively stained within 30 min. Complete staining of starved cells with a high content of intracellular neutral lipids took at least 2 h for a biomass concentration of 0.15 µL mL^−1^ (Fig. 3b). The slow staining of starved cells can be explained by different hypotheses: First, dye permeation into the starved cells could be hampered by their more rigid cell wall. Furthermore, diffusion of Nile red from the hydrophobic cell membrane via the aqueous cytoplasm towards the liposomes, as well as the dye uptake by the liposomes itself, could be rate-limiting [27, 33, 34].

However, the contribution of each possible effect to the slowed staining kinetics was not further investigated. Instead, a biovolume of 0.15 µL mL^−1^ and a staining time of 3 h were chosen as standard parameters to enable quantitative staining of cells with differing lipid content within the linear range of the assay.

**Fig. 4:**
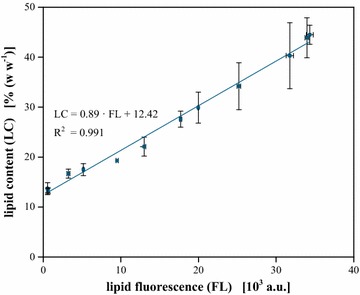
Gravimetric calibration of the lipid assay. The Nile red staining deviated biomass specific lipid fluorescence is linearly correlated with the fraction of intracellular lipids measured by extraction and subsequent gravimetric analysis. Cells with a biovolume of 0.15 µL mL^−1^ were stained for 3 h at 40 °C in 25 % (v v^−1^) DMSO with 1 mg L^−1^ Nile red. *Error bars* of the Nile red assay deviated from analytical replicates (n = 5). *Error bars* of the gravimetric measurement from technical replicates (n = 3)

To check for precision, as well as to enable absolute quantification, the improved assay was calibrated against gravimetric determination of the lipid content. Therefore, algal samples after different times of N-starvation were analysed, using both orthogonal methods (Fig. 4).

Within 0.6 × 10^3^–34.3 × 10^3^ a.u., the obtained fluorescence was found to be linearly correlated (R^2^ = 0.991) to a lipid content ranging from 13.3–44.5 % (w w^−1^). Average relative errors were ±2 and ±8 % for lipid fluorescence and gravimetry, respectively.

Resulting, fluorescence data from Nile red staining can be assessed for absolute quantification of the cellular lipid content, while accuracy is significantly (p < 0.05) enhanced compared to gravimetric measurement. Key to success enabling reliable and valid quantitative data obtained from the fluorescence assay is the standardized approach with fixed values for biomass concentration (i.e. biovolume instead of optical density), Nile red concentration and incubation conditions.

Strikingly, analysing samples with minimal lipid content generated by harvest of exponentially growing cultures revealed a fluorescence signal in the range of the background signal, i.e. no lipids were detected in the cells, whereas gravimetric analysis showed a lipid content of 13.3 % (w w^−1^). This offset may be explained by the differing specificity of the methods. By choosing an excitation/emission setup of 480/570–580 nm, Nile red staining was designed to specifically stain neutral lipids [33] which are typically produced during nutrient starvation. On the contrary, gravimetric measurement is based on the extraction of the total cellular lipids [16]. Besides neutral lipids, these additionally contain e.g. the membrane phospholipids that are not detected by the Nile red staining in this case. This fraction is represented by the y offset of the correlation function (Fig. 4) and the observed lipid content of 13.3 % (w w^−1^) is in exact agreement with previous literature reports about the biomass composition of growing *Chlorella* [41].

### Automation

**Fig. 5:**
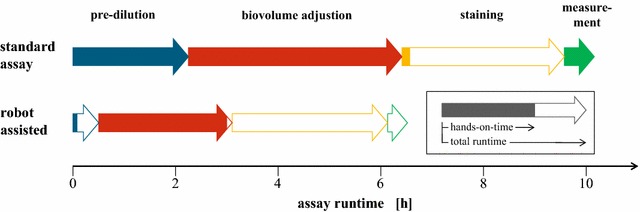
Assay automation significantly enhances analytical throughput. Using the described liquid handling platform, hands-on-time and total runtime can be reduced by 66 and 37 %, respectively. *Arrows* represent the duration of individual assay steps, while the respective *coloured fractions* represent hands-on-times. *Blue* pre-dilution of samples, *red* measurement and adjustion of biovolume, *yellow* staining, *green* sample transfer and fluorescence measurement

With respect to the increasing number of samples that can be generated from current and next generation parallelized microscale photobioreactors, not only higher accuracy and precision, but also further acceleration of lipid quantification is needed to prevent analytics from becoming a bottleneck. For this purpose, the developed assay was transferred to an established liquid handling platform [42] (Fig. 5).

Assay automation resulted in a considerable increase of experimental throughput. The workflow was designed to handle up to 48 samples per run. For this, a total runtime of the robotic assay of ca. 6.5 h, including 2.5 h of hands-on-time is needed. Compared to manual assay preparation taking 10.5 h, including 7.5 h of hands-on-time, the overall time needed was reduced by 37 %. In particular, hands-on-time was reduced by 5 h (−66 %) minimizing the needed human operator interaction. Thereby, the use of a liquid handling robot for assay automation proved to be valuable for increasing analytical throughput.

As pipetting steps during assay preparation may have a major impact on data reliability, the effect of routinely running lipid analysis on a laboratory robot was investigated with regard to accuracy and precision within the relevant volume ranges.

**Fig. 6:**
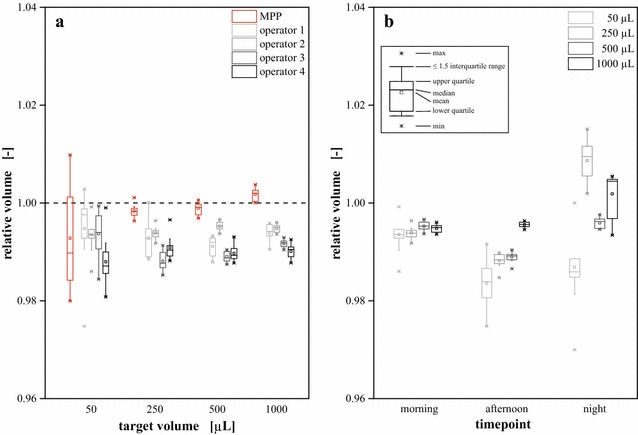
Compared to preparation by hand, automated liquid handling increases accuracy and precision. **a** Although liquid handling robots suffer from systematic and stochastic errors as well, these are typically constant, while manual pipetting by different operators (four persons in this case) can result in significant differences. **b** Daily operator performance can induce significant fluctuations of manual pipetting performance. Automated pipetting conducted by JANUS Integrator liquid handling platform and manual pipetting by four different operators using an Eppendorf Research Plus pipette. Volumes determined gravimetrically using a semi-micro balance and normalized to the respective target volumes. *Error bars* deviated from analytical replicates (n = 10)

Different volumes of desalted water were repeatedly transferred either by the liquid handling robot or manually by four different operators using the same equipment (Fig. 6a). At a target volume of 50 µL no significant difference between automated and manual liquid transfer was observable. On the contrary, for target volumes >50 µL the robotic platform ensured an improved performance compared to manual pipetting. With an average systematic error of 0.3 % a significantly (p < 0.05) higher accuracy was achieved than by manual pipetting (0.6–1.0 %, depending on the individual operator). Nevertheless, stochastic fluctuations were comparable between automated (0.3 %) and manual liquid transfer (0.2–0.3 %).

The daily performance for pipetting accuracy and precision was investigated as well (Fig. 6b). Depending on time (morning, afternoon, night) and target volume the errors of one individual operator suffered from noticeable fluctuations. In this case, the pipetting performance was best during the morning hours as a relatively constant systemic error of 0.6 % with stochastic fluctuations of 0.2 % on average were achieved. During progress of the day, a loss of accuracy and precision was observable as systematic and stochastic errors significantly (p < 0.05) grew up to 1.1 and 0.4 %, respectively.

It became apparent that replacing manual pipetting by a liquid handling robot increases pipetting accuracy and precision. Fluctuations by differing pipetting performance of individual operators performing the assay can be eliminated, as well as variations induced by daily fluctuation.

As described in the “Assay development and validation” Section, the applied biomass concentration has a strong impact on the measured fluorescence. Therefore, it needs to be adjusted as precisely as possible. For a final validation of the automated liquid handling, the individual fluctuations across technical replicates (n = 5) conducting the assay either by hand or running it on the robotic platform were evaluated. Average standard errors of 4.3 and 2.1 % were observed for manual and automated analysis, respectively. Thus, the previously demonstrated superior performance of the robotic system was proven to affect reproducibility in a positive manner. Besides the aspect of elevated throughput, this improvement gave further legitimation to the automation of the Nile red staining assay, especially with focus to running it as routine analytics.

## Conclusions

In this study, an automated high throughput assay for absolute quantification of intracellular lipids using *C.vulgaris* as a model organism is presented. Cells were stained using the lipid probe Nile red with DMSO as solvent facilitating dye permeation. Staining parameters were optimized towards MTP scale with regard to differentiating staining kinetics of cells at different physiological states and biomass concentrations. Up to 48 samples can be measured within 6.5 h on an automated liquid handling platform, reducing hands-on-time to a third compared to preparation by hand. The developed assay was calibrated against gravimetric lipid measurement allowing for absolute quantification simultaneously reducing analytical errors from ±8 to ±2 % on average. With respect to daily and operator-to-operator fluctuations, evidence is given that using a laboratory robot can increase accuracy and precision.

Contrary to established spectrofluorometric methods relying on optical density or cell number, here the biovolume was used to adjust for identical biomass concentration across all conducted measurements. It was shown that optical signals, as well as cell number cannot be applied for algae with complex lifecycle as is the case for *C. vulgaris*. Due to their mode of replication via vegetative autosporulation, the cells undergo morphological changes significantly altering their optical properties. Even different amounts of accumulated lipids could have an impact on refractive properties.

Besides improvements regarding accuracy and precision, as well as data reliability and analytical throughput, the newly established assay is especially suited for the analysis of samples from current and next generation microscale photobioreactor systems. Applying the presented staining technology, these small-scaled samples (ca. 1 mL) can easily be handled circumventing analytics from becoming a bottleneck. Thereby, the assay can contribute to boosting the time efficiency for setting up algae lipid production processes.

## Methods

### Chemicals, strain and media

All chemicals were obtained from Sigma Aldrich (Steinheim/Germany) or Carl Roth (Karlsruhe/Germany) and were of analytical grade. Cultivations were conducted axenically using the model algae *C. vulgaris* 211–11b [43] purchased from the Culture Collection of Algae at the University of Göttingen (SAG), Germany. For all experiments an enriched Bold’s Basal Medium [44] prepared from stock solutions with the following initial composition was used: 9.76 g L^−1^ 2–(*N*–morpholino) ethanesulfonic acid, 0.6 g L^−1^ K_2_HPO_4_, 1.4 g L^−1^ KH_2_PO_4_, 1.5 g L^−1^ NaNO_3_, 187.5 mg L^−1^ MgSO_4_ · 7 H_2_O, 6.25 mg L^−1^ NaCl, 125 mg L^−1^ CaCl_2_ · 2 H_2_O, 17.64 mg L^−1^ ZnSO_4_ · 7 H_2_O, 2.88 mg L^−1^ MnCl_2_ · 4 H_2_O, 2.4 mg L^−1^ Na_2_MoO_4_ · 2 H_2_O, 3.14 mg L^−1^ CuSO_4_ · 5 H_2_O, 0.94 mg L^−1^ CoSO_4_ · 7 H_2_O, 22.8 mg L^−1^ H_3_BO_3_, 9.96 mg L^−1^ FeSO_4_ · 7 H_2_O, 3.68 mg L^−1^ H_2_SO_4_, 100 mg L^−1^ Na_2_EDTA · 2 H_2_O, 62 mg L^−1^ KOH and 100 mg L^−1^ penicillin-G sodium salt. The pH value was set to 6.5 with 5 M NaOH.

### Strain maintenance and cultivation

Strain maintenance was conducted via cryopreservation of glucose-adapted cells. Phototrophic re-adaptation and biomass formation was realized by phototrophic shaking flask cultivation as described in detail in Morschett et al. [45]. Main cultures for the generation of biomass containing different amounts of lipids were conducted in shaking flasks in the same way as pre-cultures. Instead of inoculation from cryocultures, they were inoculated from exponentially growing pre-cultures to an initial biovolume of 0.1 µL mL^−1^. Different lipid contents were obtained by harvesting the cells either during growth or at different times during N-starved lipid accumulation.

### Biomass detection

As an indicator for growth, the optical density (OD) was measured by light extinction using an UV-1800 photometer (Shimadzu, Duisburg/Germany). In order to avoid interference with algal pigments, measurement was done at a wavelength of 750 nm as recommended by literature [46]. If needed, the samples were diluted using 0.9 % (w v^−1^) NaCl solution until the measured OD_750_ was within the linear range of the photometer (0.1–0.3). The samples were analysed in 10 mm polystyrene semi-micro cuvettes (ratiolab, Dreieich/Germany) while desalted water served as a blank.

Cell counts and biovolume were determined using a MultiSizer 3 (Beckman Coulter, Krefeld/Germany) particle counter equipped with a 30 µm capillary in volumetric control mode. The cell counter uses the “Coulter principle” [47]: A defined volume (100 µL) of the sample is sucked through a capillary while a constant current of 40 µA is applied between the capillary and a counter electrode. Cells passing the capillary pore displace a distinct volume of electrolyte which is corresponding to their own volume, the biovolume. As intact cells are electrical isolators due to their cell membrane, the conductivity between the capillary and the counter electrode is changed while passing the pore. Each of these events is detected as a distinct particle count. Moreover, the measured change in conductivity is directly proportional to the (bio)volume of the particle passing the pore. From the biovolume of each individual cell, the diameter is calculated assuming a spherical shape. Thus, the particle size or biovolume resolved particle count distribution is deviated. The cells were diluted below OD_750_ = 0.025 in CASYton buffer (Schärfe Systems, Reutlingen/Germany) and only particles sizing from 1.8 to 14 µm were analysed. The detection of cells smaller than 1.8 µm is distorted by cell debris which are in the same size. However, *C.vulgaris* is typically bigger than 2 µm. Only few cells are of the same size as the cell debris. In comparison to the majority of cells ranging from 3 to 10 µm, the smaller cells have a significantly smaller volume. In fact, the measured biovolume of the cell debris and of the “mini cell” fraction (<1.8 µm) is typically in the range of <1 % of the fraction ranging from 1.8 to 14 µm and can thus be neglected without introducing significant error in biovolume quantification.

### Gravimetric lipid quantification

Gravimetric quantification of cellular lipid content was carried out via a modified single-step extraction method, as reported by Axelsson and Gentili [16]. An aliquot of 40 mL cell suspension was filled into a 50 mL tube, centrifuged for 10 min at 3939 × g and 4 °C in a Labofuge 400R (Heraeus, Hanau/Germany) and the supernatant was discarded. After resuspending the pellet in 40 mL 0.9 % (w v^−1^) NaCl solution, centrifugation was repeated and the resulting supernatant was discarded once more. The cell pellet was lyophilized using an LT–105 freeze dryer (Christ Gefriertrocknungsanlagen, Osterode am Harz/Germany) until constant weight. The freeze-dried biomass was homogenized using a spatula and a known aliquot of ≤100 mg was filled into a 50 mL tube. After addition of 14 mL of chloroform and 7 mL of methanol, the lipids were extracted at 60 °C for 1 h in a Multitron Standard shaking incubator (Infors HT, Einsbach/Germany) at 250 rpm and a shaking diameter of 25 mm. 5.6 mL of 0.73 % (w v^−1^) NaCl solution were added and vigorously mixed. The resulting phases were separated by centrifugation for 2 min at 3939 × g and 4 °C in a Labofuge 400R (Heraeus, Hanau/Germany). The lower chloroform-phase was quantitatively collected and filled into a dried and pre-weighed 15 mL tube. The lipid extract was evaporated at 40 °C until constant weight using a T6120 drying cabinet (Heraeus, Hanau/Germany). After cooling to room temperature in a desiccator and weighing the extract, the lipid content (LC) was calculated according to Eq. (1):

1 $$LC = \frac{{\left( {m_{b} - m_{n} } \right)}}{{m_{B} }} \cdot 100^{{}}\, \%$$

with m_b_ being the brutto tube weight [mg], m_n_ the netto tube weight [mg] and m_B_ the amount of extracted biomass [mg].

### High throughput lipid quantification

For the quantification of intracellular neutral lipid content at high throughput, a modified version of the Nile red-based fluorometric assay described by Chen et al. [33] was used at 96 well MTP format. To enable an elevated throughput, the assay was automated using an established laboratory robotic platform [42, 48]. The setup consisted of a JANUS Integrator liquid handling platform (Perkin Elmer, Rodgau/Germany) equipped with a Varispan liquid handling arm and an MTP railgripper (a robotic arm that is designed to transfer MTPs across the deck of the liquid handling robot) with integrated Teleshake95 MTP shaker/heater (Inheco, Martinsried/Germany) and an EnSpire MTP photometer (Perkin Elmer, Rodgau/Germany). In this context, the assay was designed to be capable of handling up to 48 samples per run. An illustration of the robotic setup is given in the additional material (Additional file 1).

**Fig. 7:**
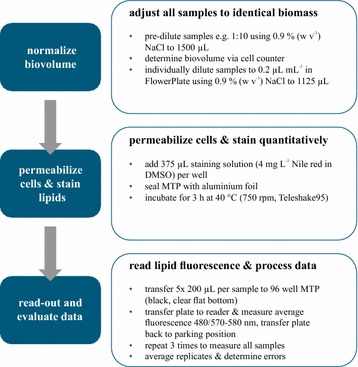
Schematic representation of the workflow for automated quantification of intracellular neutral lipids at high throughput. The protocol was designed to run on a JANUS Integrator liquid handling platform ensuring minimized hands-on-time

After initial determination of the biovolume of the respective sample, all subsequent steps were carried out using the robotic platform. Each sample was individually diluted to a biovolume of 0.2 µL mL^−1^ using 0.9 % (w w^−1^) NaCl in an 48 well flower shaped MTP (m2p-labs, Baesweiler/Germany) resulting in a total volume of 1175 µL. 375 µL of a freshly prepared staining solution (4 mg L^−1^ Nile red in DMSO) were added to each diluted sample, the plate was sealed with a self-gluing aluminium foil (Greiner Bio-One, Solingen/Germany) and incubated at 40 °C and 750 rpm on the Teleshake95 for 3 h. Afterwards, the stained samples were subsequently transferred to three black 96 well MTPs with clear polystyrene F-bottom (Greiner Bio-One, Solingen/Germany) in five 200 µL aliquots per sample. During this step, the samples were continuously shaken to ensure proper mixing. After the transfer of samples to a 96 well MTPs, an equivalent volume of water was filled into the emptied wells to keep the FlowerPlate tared during shaking. The plates were successively transferred to the EnSpire photometer and internally shaken for another 15 s at 600 rpm and a shaking diameter of 1 mm in orbital mode. After excitation at 480 nm fluorescence in the range of 570–580 nm was measured and the average value across the spectrum was calculated for each individual well. Transferring the samples to the 96 well MTP, taring of the FlowerPlate and measuring with the EnSpire photometer were scheduled to minimize the workflow runtime. Figure 7 gives a schematic overview of the complete assay.

### Pipetting accuracy and precision

Pipetting accuracy (systematic errors) and precision (stochastic errors) were determined gravimetrically, as previously described in literature [49]. Different amounts of desalted water were transferred to pre-dried (48 h, 80 °C, cooled to room temperature in a desiccator) glass vials and quantified via a CPA225D semi micro balance (Sartorius, Göttingen/Germany). For manual pipetting, 100 and 1000 µL Research^®^ Plus pipettes (Eppendorf, Hamburg/Germany) were used, while automated liquid handling was performed by the JANUS Integrator robotic system in single dispense per aspirate mode. All pipetting steps were repeated in 10 individual replicates. Accuracy and precision were calculated according to Eqs. (2) and (3):

2 $$accuracy = n^{ - 1} \cdot \sum\limits_{i = 1}^{n} {\left| {m_{i} - m_{t} } \right|} \cdot 100^{{}}\, \%$$

3 $$precision = \frac{{\frac{{\sqrt {\sum_{i = 1}^{n} {\left( {\left| {m_{i} - m_{t} } \right| - n^{ - 1} \cdot \sum_{i = 1}^{n} {\left| {m_{i} - m_{t} } \right|} } \right)^{2} } } }}{n - 1}}}{{n^{ - 1} \cdot \sum_{i = 1}^{n} {m_{i} } }} \cdot 100^{{}}\, \%$$

with m_i_ being the amount of transferred water [mg], m_t_ the corresponding target amount to be transferred [mg] and n the number of replicates [-].

### Statistical analysis

For all statistical analyses, two-sided t-tests for unequal variances (95 % significance level) were applied using Origin9.1.0G (OriginLab Corporation, Northampton/United States).

## Additional file

10.1186/s12934-016-0433-7 Illustration of the robotic platform with focus on deck layout.

## Abbreviations

DMSO: dimethyl sulfoxide
FL [10^3^ a.u.]: lipid fluorescence
LC [% (w w^−1^)]: lipid content
m_b_ [mg]: brutto tube weight
m_B_ [mg]: mass of extracted biomass
m_i_ [mg]: transferred amount of water
m_n_ [mg]: netto tube weight
m_t_ [mg]: target amount of water to be transferred
MTP: microtiter plate
n [-]: number of replicates
OD_*i*_ [-]: optical density at a wavelength of *i* nm
