# Supplementary figures and images for: Automation of a Nile red staining assay enables high throughput quantification of microalgal lipid production
Source: Microb Cell Fact. 2016 Feb 9;15:34. doi: 10.1186/s12934-016-0433-7 (PMC4748563; doi:10.1186/s12934-016-0433-7)

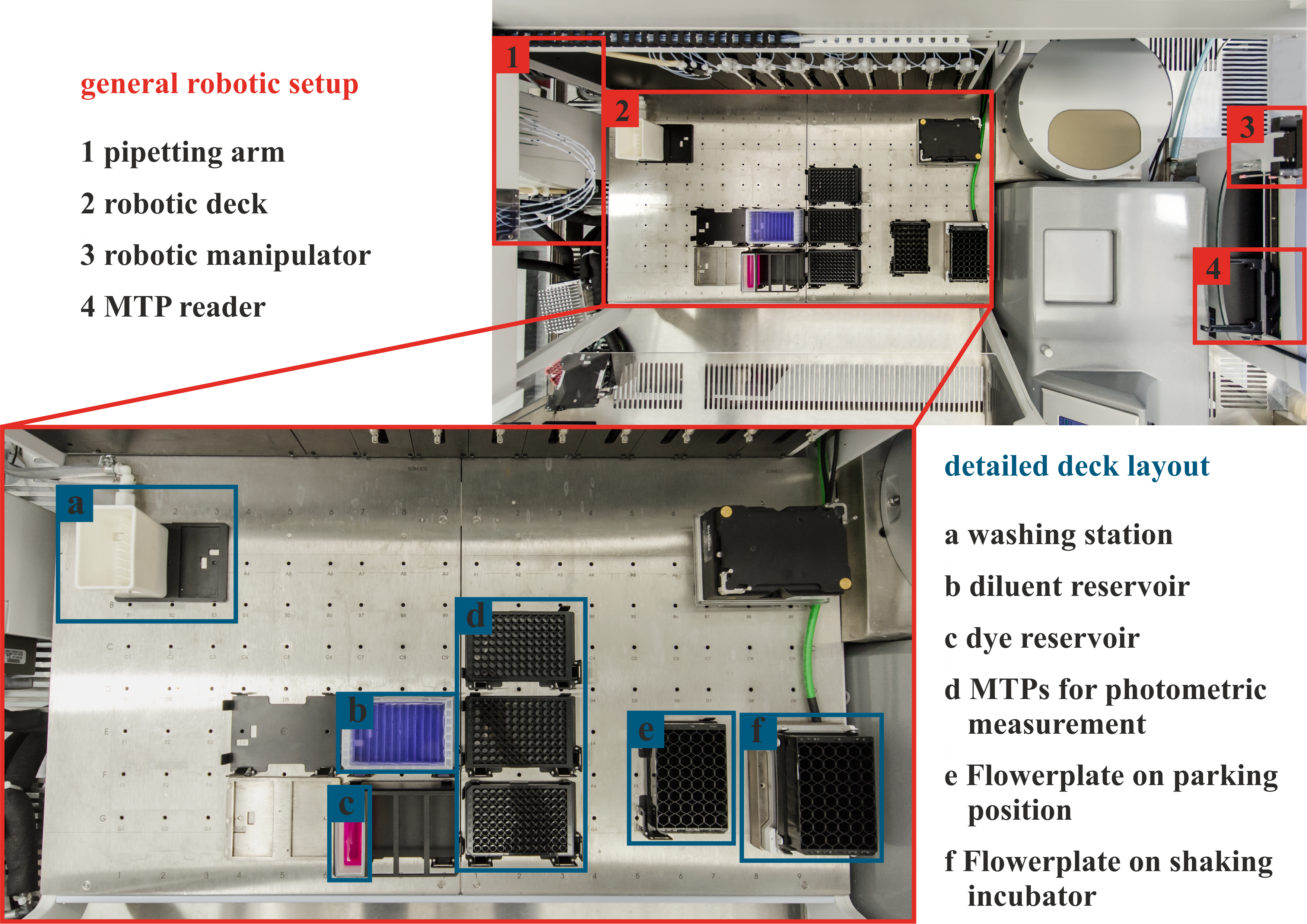

Supplement: Supplementary file 1 — 10.1186/s12934-016-0433-7 Illustration of the robotic platform with focus on deck layout. [file 12934_2016_433_MOESM1_ESM.png]
